# Clinical Features of COVID-19 Patients in Jordan: A Study of 508 Patients

**DOI:** 10.2174/1874306402115010028

**Published:** 2021-06-18

**Authors:** Mahmoud Al-Balas, Hasan I. Al-Balas, Rami Alqassieh, Hamzeh Al-Balas, Almu'atasim Khamees, Rahaf Al-Balas, Samir Al-Balas

**Affiliations:** 1 Department of General and Special Surgery, Faculty of Medicine, Hashemite University, Zarqa, Jordan; 2 Faculty of Medicine, Yarmouk University, Irbid, Jordan; 3 Department of General and Special Surgery, Faculty of Medicine, Hashemite University, Zarqa, Jordan; 4 Faculty of Medicine, Yarmouk University, Irbid, Jordan; 5 Department of Basic Sciences, Faculty of Medicine, Yarmouk University, Irbid, Jordan

**Keywords:** SARS-CoV-2, COVID-19, Hospitalization, Medical comorbidities, Symptomatic patients, Asymptomatic patients

## Abstract

**Background:**

The symptoms of COVID-19 have a wide range of severity ranging from no symptoms at all to mild symptoms, such as fever, cough, sore throat, general weakness. Moreover, in some situations, patients may develop severe complications as pneumonia, and sepsis, leading to death. This study aims to investigate the characteristic features of COVID-19 patients based on their medical condition prior to COVID-19 diagnosis.

**Methods:**

A retrospective cohort study took place between the 1st of April 2020 and the 31^st^ of June 2020 in Prince Hamzah Hospital, Jordan. Patients were diagnosed by the Real-Time Reverse Transcriptase (RT)–PCR Diagnostic Panel, either through screening or for those who developed symptoms. During this period, patients who tested positive for COVID 19 were admitted to the hospital regardless of their symptoms according to the local government health policies. A total of 508 Patients were involved and divided into two groups based on the presence or absence of chronic illnesses prior to COVID-19 diagnosis.

**Results:**

A total of 371 patients were medically free (220 males and 151 females). Among them, 153 patients were symptomatic (41.2%), with an average hospitalization of 18 days. Generalized malaise, dry cough, and fever were the most common reported symptoms (51%, 45.8%, and 41.8%, respectively). On the other hand, the total number of COVID-19 patients with predefined comorbidities was 137 (93 males and 44 females). Among them, 86 patients (62.8%) were symptomatic, with an average duration of admission of 19.3 days. Similar to medically free patients, dry cough, generalized malaise, and fever were the most commonly reported symptoms (50%, 43%, and 38.4%, respectively). There was a statistically significant correlation between the presence of chronic illnesses and the development of symptoms among COVID-19 patients (*P* = 0.0001).

**Conclusion:**

Dry cough, generalized malaise, and fever were the most commonly reported symptoms among our patients regardless of their medical condition. The average duration of hospitalization in medically free patients was less than patients with comorbidities, and it was less among asymptomatic compared to symptomatic patients. More than half of our COVID-19 patients were male and asymptomatic. A significant correlation between patients' medical condition and the possibility of developing symptoms in response to COVID-19 was identified.

## 1 INTRODUCTION

On December 8, 2019, the China Health Authority reported several cases of unknown etiology pneumonia discovered in Wuhan city of Hubei Province, China. After that, the China Health Authority alarmed the World Health Organization (WHO) about the outbreak on December 31, 2019 [1]. The scientists confirmed that these outbreak pneumonia cases were caused by a new coronavirus called Severe acute respiratory syndrome coronavirus 2 (SARS-CoV-2) that transmit from human to human and had a rapid growth reaching many countries, causing the third coronavirus epidemic in the 21^st^ century after Severe Acute Respiratory Syndrome (SARS) and Middle East Respiratory Syndrome (MERS). On January 30, 2020, the WHO declared a Public Health Emergency of International Concern (PHEIC) [2] and a pandemic on March 11, 2020 [3].

In Jordan, the first confirmed case of COVID-19 was reported on March 2, 2020 [4] of a Jordanian male who came from Italy. On March 15, 2020, few cases were additionally diagnosed with a total number of 12 cases [4]. The number of cases had increased progressively but slowly since that time. On April 12, 2020, the total confirmed cases were only 389, thus, WHO classified Jordan as having a “cluster of cases” transmission for SARS-CoV-2 [5]. Jordanian authorities have announced a state of emergency and declared curfew and lockdown across the country to control disease transmission. Health authorities have adopted quarantine in governmental-sponsored facilities followed by 7 to 24 days of home quarantine. By the end of August 2020, the total number of COVID-19 cases in Jordan reached a total of 2034 cases and 15 cases of COVID-19 related deaths. The global progression of infected cases suggests that this virus can be transmitted by an asymptomatic carrier [6]. However, the symptoms of COVID-19 appear after exposure to the virus by 2-14 days along with a wide range of severity from no symptoms at all to mild symptoms like fever, cough, sore throat, general weakness, and in some cases with severe symptoms like severe pneumonia, severe inflammatory response and secondary sepsis, and death [7, 8]. The severity of symptoms depends on many factors, including older ages [7, 8], gender [8, 9], smoking [9, 10], body temperature [10], elevated albumin and CRP [10], having an underlying medical condition like hypertension and DM [7, 8], all these factors lead to more severe symptoms and high risk for Intensive Care Unit (ICU) admission.

In this study, we aimed to investigate the characteristic features of COVID-19 patients in relation to their medical condition. Patients’ gender, age range, mean age, duration of hospitalization, and the common presenting symptoms were analyzed in correlation with patients' medical status. As a secondary goal, we tried to explore the association between smoking and the development of symptoms among COVID-19 patients.

## 2 METHODS

This retrospective cohort study assessed COVID-19 patients who were hospitalized during the period between the 1^st^ of April 2020 and the 31^st^ of June 2020 in Prince Hamza Hospital (PHH) in Jordan. PHH is considered a tertiary referral hospital and one of the main centers dedicated to COVID-19 patients.

Patients were diagnosed by Real-Time Reverse Transcriptase (RT)–PCR Diagnostic Panel, either through screening or developed symptoms. According to the local government health policies, patients who tested positive for COVID 19 during this period were admitted to the hospital regardless of their symptoms.

A total of 550 patients were diagnosed positive for COVID 19 during that period. Patients who were less than 6 years (n=42) were excluded from the study to achieve realistic data regarding subjective patient symptoms. The remaining 508 Patients were divided into two groups depending on the presence or absence of chronic illnesses such as Diabetes Mellitus (DM), Hypertension (HTN), Ischemic Heart Disease (IHD), tumors, autoimmune diseases, asthma, and solid and/or bone marrow transplant.

### 2.1 Statistical Analysis

Data were recorded in a Microsoft Excel (Redmond, WA, USA) spreadsheet and analyzed by SPSS program version 16.0. Statistical significance was assessed using a two-tailed Fisher’s exact test (statistical significance was considered for *P*< 0.05).

## 3 RESULTS

In this study, a total of 508 patients with COVID-19 were included (313 males and 195 females). Patients with a diagnosis of COVID-19 were categorized based on their medical history into either medically free patients (n=371) or patients with predefined comorbidities (n=137).

Based on gender, 138 male patients were symptomatic (44.1%) in comparison to 101 females (51.8%), no statically significant correlation between gender and development of symptoms was identified (*P*= 0.1002) (Table **1**).

**Fig. (1):**
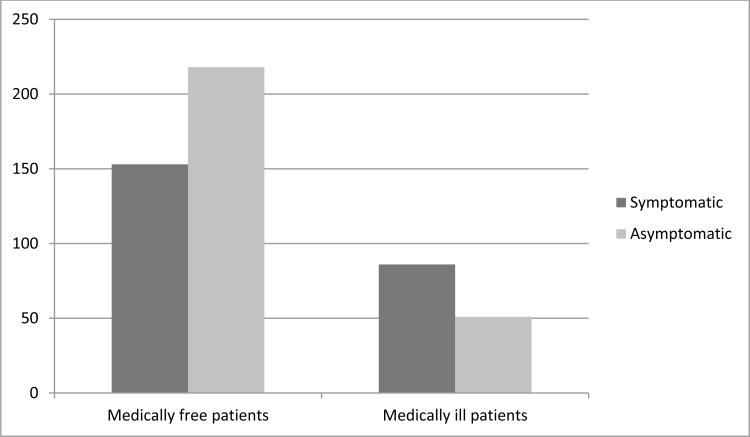
COVID-19 related symptoms in correlation with predefined medical condition.

Among medically free patients (n=391), only 153 patients were symptomatic (39.1%). This figure was statistically less compared to patients with predefined comorbidities, where approximately 62.7% of them were symptomatic (*P*=0.0001) (Fig. **1**).

### 3.1 Medically Free Patients with a Diagnosis of COVID-19

The total number of patients was 371 (220 males, 151 females). Patients' ages ranged between 6 and 74 years (Mean 30 ±14.3). Among those, 153 patients were symptomatic (41.2%), and an average duration of hospitalization was 18 days. On the other hand, 218 patients were asymptomatic (58.8%), and they were diagnosed during screening. Those patients had an average duration of stay in the hospital of 14 days. The overall average duration of stay for all medically free patients (n=371) was 15.5 days (Table **1**).

Regarding the COVID-19 symptomatic patients, generalized malaise, dry cough, and fever were the most common reported symptoms (51%, 45.8%, and 41.8%, respectively). Among symptomatic males, the dry cough was the most common symptom, while generalized malaise was the most common among females. Moreover, patients also reported other symptoms such as headache (31.4%), chills and rigors (28.1%), myalgia (21.6%), diarrhea (20.3%), sweating (15%), wet cough (14.4%), abdominal pain (11.1%), chest pain (9.2%), palpitations (5.2%), shortness of breath (17.6%), and one patient presented with hemoptysis. The prevalence of nasal congestion, loss of smell, loss of taste, and rhinorrhea were 32.7%, 32%, 26.8%, 24.2%, respectively (Table **2**).

Regarding the patient history of smoking, only 61 patients within the medically-free group were smokers (16.4%). Even though it was not statistically significant, the prevalence of COVID-19 related symptoms was lower among non-smokers than smokers within this group of patients (39.7% *Vs*. 49.2%) (*P* = 0.2) (Table **3**).

### 3.2 Chronic Medical Ill Patients with a Diagnosis of COVID 19

The total number of patients was 137 (93 males, 44 females). Patients' ages ranged between 14 and 87 years (Mean 53 ±15.8). Hypertension, diabetes, and cardiovascular disease were the most frequent comorbidities, with a prevalence of 62%, 51%, and 16%, respectively. A total of 62 patients (45.2%) reported more than one comorbidity (Table **4**).

Among this group, the majority of patients were symptomatic (n=86; 62.8%), with an overall average duration of hospitalization of 17 days. Symptomatic patients had a longer duration of hospitalization than non-symptomatic patients (19.3 *Vs*. 16.9 days). A total of 21 patients required admission to the Intensive Care Unit (ICU), and 4 patients died during their admission secondary to respiratory failure (Table **1**).

Similar to medically free patients, dry cough, generalized malaise, and fever were the most commonly reported symptoms (50%, 43%, and 38.4%, respectively). Other reported symptoms were headache (25.6%), chills and rigors (33.7%), myalgia (27.9%), diarrhea (23.3%), sweating (11.6%), wet cough (19.8%), abdominal pain (12.8%), chest pain (12.8%), palpitations (3.5%), shortness of breath (27.9%), and also one patient presented with hemoptysis. Sinonasal symptoms were less frequent in this group compared to medically free patients. Nasal congestion, loss of smell, loss of taste, and rhinorrhea were presented in 17.4%, 18.6%, 16.3%, and 18.6% of the patients, respectively (Table **5**). Similar to medically free patients, there was no correlation between the history of smoking and the development of symptoms among patients in this group (Table **3**).

## 4 DISCUSSION

In this study, the mean age of COVID-19 medically free patients was 30 years and 53 years for chronic medically ill patients with an overall mean of 36.2 years, which is younger than reported by Jiang *et al*., Chen *et al*., and Yang *et al*. [12-14] (51.2, 55.5 and 59.7 sequentially). According to the 2019 report of the Jordanian department of statistics, 70.5% of the Jordanian population is younger than 35 years [15-18]. Thus, Jordan is a young community, and this may be the main cause for this variation in the mean ages of patients. Moreover, 59.3% of medically free patients and 67.1% of chronic medically ill patients were males. This is consistent with many studies' findings that reported that males more commonly develop COVID [19, 8, 9, 11]. The mortality rate was higher in males than females, where three out of four patients with COVID-19 related deaths were males. Our finding was consistent with Jin *et al*. [12] who stated that males mortality from COVID-19 was 2.4 fold higher than females. In our study, mortality among chronically ill patients was reported in patients with HTN and IHD. The underlying mechanisms behind the poor prognosis of hypertensive COVID-19 patients are not well known. Activation of the renin-angiotensin system (RAS) in a hypertensive patient may contribute to lung injury among COVID-19 patients by promoting an inflammatory response (cytokine storm) [29].

The average duration of hospital stay in medically free patients was 15.5 days, during 17 days in medically ill patients and 15.9 days for overall patients. However, Jiang *et al*. [13] reported that the average duration of hospitalization was 16.6 days, whereas 18.2 in Qin *et al*. [16]. Moreover, in this study, we investigated the average duration of stay between symptomatic and asymptomatic patients, and we found that the average duration among those two groups was 17.7 days in symptomatic patients and 14.5 days in asymptomatic patients.

Approximately 58.8% of medically free patients and 37.2% of medically ill patients were asymptomatic with an overall rate of 53% among all patients; this was higher than figures reported by Nishiura *et al*. [17], Mizumoto *et al*. [18], Qin *et al*. [16], and Chinese CDC [19] findings (30.8%, 17.9%, 13.7%, and 1.2% sequentially).

In comparison with symptoms reported in the literature, 48.1% of our patients had generalized malaise, which was comparable to Huang *et al*. [20] findings (44%) and Xu *et al*. [21] findings (52%). Moreover, it was less than Wang *et al*. [22] (69.6%) and more than Qin *et al*. 16 (14.5%). Regarding the cough, 47.3% of patients had dry cough compared to 59.4% from Wang *et al*. [22] patients. Overall, 63.6% of our patients had an either dry or wet cough, which is lower than Huang *et al*. [20], Xu *et al*. [21], and Chen *et al*. [11] (76%, 81%, 82% sequentially) and higher than Qin *et al*. [16] (37.7%). Fever was reported by 40.6% of our patients, which is lower than Qin *et al*. [16], Xu *et al*. [21], Chen *et al*. [11], Huang *et al*. [20], and Wang *et al*. [22] findings (56.5%, 77%, 83%, 98%, 98.6% sequentially).

Recent studies stated that loss of smell “anosmia” and loss of taste “dysgeusia” were more common among COVID-19 patients by 28.6 fold [23] and is considered an important symptom of COVID-19 infection and maybe the only presentation without other symptoms [24]. In our results, loss of smell was approximately 1.7 folds more in medically free patients than medically ill patients (32%, 18.6%, respectively). Likewise, loss of taste was 1.6 folds more in medically free patients than ill patients (20.8%, 16.3%, respectively). Female patients were 2 times more likely to experience anosmia than male patients (18.5%, 9.3% respectively) and 2.2 times to have dysgeusia, a loss of taste than males. Similarly, Lee *et al*. [25] and Spinato *et al*. [26] reported that anosmia and dysgeusia were more frequent in females.

Out of all patients, the percentage of patients who have symptoms was more in medically ill patients (62.8%) than medically free patients (41.2%). Regarding the medically ill patients (n=137), 21 of them (15.3%) required an ICU admission with a mortality of 2.9%. Many studies reported that patients with severe COVID-19 outcomes who had severe symptoms or were treated in the ICU were older and with comorbidities [7-9, 11, 14, 22].

Our findings showed no significant correlation between the history of smoking and the development of symptoms in relation to patient medical conditions. Similar findings were reported by other researchers [14, 20, 27]. On the other hand, Sanchez-Ramirez *et al*. [9] and Patanavanich *et al*. [28] reported that the progression of severe symptoms or outcomes was more among smokers by 1.98 and 1.91 folds, respectively. Also, Liu *et al*. [10] stated that smoking is a risk factor for disease progression.

## CONCLUSION

Dry cough, generalized malaise, and fever were the most commonly reported symptoms regarding our patients in medically free and medically ill patients. The average duration of hospital stay in medically free patients was less than medically ill, and was less in asymptomatic compared to symptomatic patients. More than half of our patients were asymptomatic and male. There was a statistically significant correlation between the presence of chronic illnesses and the development of symptoms in COVID-19 patients.

## Figures and Tables

**Table 1:** The distribution of patients regarding symptoms, gender, and health status.

-	**Symptomatic**		**Asymptomatic**		**Total**
	**Male**	**Female**	**Male**	**Female**	
Medically free patients	79	74	141	77	371
Medically ill patients	59	27	34	17	137
Total	138	101	175	94	508
Total	239		269		508

**Table 2:** The presenting symptoms of COVID 19 in medically free patients.

**Symptom**	**Male**	**Female**	**Total**	**Percentage**
Dry Cough	39	31	70	45.8%
Fever	32	32	64	41.8%
Wet Cough	12	10	22	14.4%
Chills/Rigors	14	29	43	28.1%
Sweating	6	17	23	15%
Generalized Malaise	35	43	78	51%
Myalgia	7	26	33	21.6%
Shortness of Breath	9	18	27	17.6%
Headache	17	31	48	31.4%
Hemoptysis	0	1	1	0.7%
Diarrhea	10	21	31	20.3%
Chest Pain	6	8	14	9.2%
Abdominal Pain	6	11	17	11.1%
Palpitations	0	8	8	5.2%
Loss of Taste	16	25	41	26.8%
Loss of Smell	19	30	49	32%
Nasal Congestion	20	30	50	32.7%
Rhinorrhea	14	23	37	24.2%

**Table 3:** The relation between smoking and the presence of symptoms in COVID 19 patients.

**Medical Free Patients**			
-	**Symptomatic**	**Asymptomatic**	**Total**
Smoker	30	31	61
Non-smoker	123	187	310
Total	153	218	371
**Medically Ill Patients**			
Smoker	13	6	19
Non-smoker	73	45	118
Total	86	51	137

**Table 4:** Comorbidities among COVID 19 patients (n = 137).

**Comorbidities**	**Number**	**Percentage**
HTN*	85	62%
DM*	70	51%
IHD*	22	16%
HYPOTHYROID	18	13%
ASTHMA	12	8.7%
PROSTATE HYPERPLASIA	6	4.3%
AUTOIMMUNE DISEASE	4	2.9%
MIGRAINE	4	2.9%
CANCER	3	2.1%
COPD*	3	2.1%
RENAL FAILURE	1	0.7%
CVA*	1	0.7%

(HTN: Hypertension; DM Diabetes Mellitus; IHD Ischemic Heart Disease; COPD Chronic Obstructive Pulmonary Disease; CVA Cerebrovascular accident.

**Table 5:** The presenting symptoms of COVID 19 in chronic medical ill patients.

**Symptom**	**Male**	**Female**	**Total**	**Percentage**
Dry Cough	29	14	43	50%
Fever	25	8	33	38.4%
Wet Cough	12	5	17	19.8%
Chills/Rigors	18	11	29	33.7%
Sweating	7	3	10	11.6%
Generalized Malaise	23	14	37	43%
Myalgia	14	10	24	27.9%
Shortness of Breath	12	12	24	27.9%
Headache	13	9	22	25.6%
Hemoptysis	1	0	1	1.2%
Diarrhea	12	8	20	23.3%
Chest Pain	7	4	11	12.8%
Abdominal Pain	8	3	11	12.8%
Palpitations	2	1	3	3.5%
Loss of Taste	7	7	14	16.3%
Loss of Smell	10	6	16	18.6%
Nasal Congestion	9	6	15	17.4%
Rhinorrhea	9	7	16	18.6%
